# Synergistic Enhancement of Visible-Light-Driven Photocatalytic H_2_O_2_ Production over g-C_3_N_4_/ZnCdS by Zn Vacancies and Heterointerface Engineering

**DOI:** 10.3390/nano16080484

**Published:** 2026-04-18

**Authors:** Zhenyu Wang, Wei Yan, Yingcong Wei, Jing Xu, Yuee Xie, Yuanping Chen, Xiaohong Yan

**Affiliations:** 1 School of Physics and Electronic Engineering, Jiangsu University, Zhenjiang 212013, China; 2212326003@stmail.ujs.edu.cn (Z.W.); wyan@ujs.edu.cn (W.Y.); ycwei@ujs.edu.cn (Y.W.); chenyp@ujs.edu.cn (Y.C.); 2 Quantum Sensing and Agricultural Intelligence Detection Engineering Center of Jiangsu Province, Zhenjiang 212013, China; yanxh@njupt.edu.cn; 3 School of Science, Nanjing University of Posts and Telecommunications, Nanjing 210023, China

**Keywords:** hydrogen peroxide, photocatalysis, g-C_3_N_4_, Zn vacancies, Z-scheme heterojunction

## Abstract

Hydrogen peroxide (H_2_O_2_) is an important green oxidant, and developing efficient visible-light-driven routes for its synthesis is highly desirable. Herein, a CN/Zn_V_-ZCS composite photocatalyst was constructed by coupling g-C_3_N_4_ (CN) with Zn-vacancy-containing ZnCdS (Zn_V_-ZCS) for photocatalytic H_2_O_2_ production. The optimized CN/Zn_V_-10 delivered 44.58 mmol g^−1^ H_2_O_2_ within 60 min under 425 nm LED irradiation, outperforming pristine CN, ZCS, Zn_V_-ZCS, and vacancy-free CN/ZCS, with good cycling stability. Trapping and EPR results identify O_2_ as the key electron acceptor and ·O_2_^−^ as an important intermediate. Structural characterization and XPS results indicate successful Zn-vacancy introduction, intimate heterointerface formation, and interfacial electron redistribution. Combined VB-XPS, photoelectrochemical, and reactive-species analyses suggest that Zn vacancies are favorable for O_2_ adsorption/activation, whereas the CN/Zn_V_-ZCS heterointerface promotes charge separation and migration. Based on the available evidence, a Z-scheme interfacial charge-transfer pathway is established in the CN/Zn_V_-ZCS system.

## 1. Introduction

Hydrogen peroxide (H_2_O_2_) is an important green oxidant and high-value-added chemical with wide applications in environmental remediation, organic synthesis, and sterilization [1,2,3,4,5]. At present, industrial H_2_O_2_ production mainly relies on the anthraquinone process. However, this process generally involves a complicated procedure, relatively high energy consumption, and potential risks during storage and transportation [1,2,3,5]. Therefore, the development of green, mild, and sustainable routes for H_2_O_2_ synthesis is of considerable scientific and practical importance [1,2,3,4,5,6].

In recent years, photocatalytic H_2_O_2_ production from water and O_2_ under ambient conditions has attracted increasing attention because of its mild reaction conditions, environmental compatibility, and potential for solar-energy utilization [1,2,3,4,5,6,7]. Nevertheless, the practical efficiency of current photocatalytic systems is still limited by several factors [2,3,6]. On the one hand, the adsorption and activation of O_2_ are often insufficient, which restricts the subsequent reduction process. On the other hand, photogenerated electron-hole pairs tend to recombine rapidly, resulting in low charge-utilization efficiency. In addition, the generated H_2_O_2_ may undergo further decomposition during the reaction, which also lowers the overall yield [2,3,5,6]. Therefore, simultaneously improving O_2_ activation and charge separation/utilization remains a key issue in the development of efficient photocatalytic H_2_O_2_ production systems [1,2,3,4,5,6].

As a typical metal-free polymeric semiconductor, g-C_3_N_4_ has received continuous attention in photocatalytic H_2_O_2_ production because of its facile preparation, good chemical stability, and visible-light response [8,9,10]. Previous studies have shown that surface regulation, structural optimization, and interfacial modification can improve the light-harvesting ability, charge-separation efficiency, and O_2_ reduction behavior of g-C_3_N_4_ to some extent [8,9,10,11,12,13,14,15,16]. However, the performance of pristine g-C_3_N_4_ is still restricted by its intrinsically low conductivity, strong excitonic effect, and sluggish interfacial reaction kinetics [9,10,11,12,13,14,15,16]. In comparison, ZnCdS-based sulfides possess relatively narrow band gaps and strong visible-light absorption, making them promising candidates for photocatalytic reduction reactions [17,18]. In particular, introducing Zn vacancies is expected to regulate the local coordination environment and surface electronic structure, thereby providing more favorable conditions for O_2_ adsorption/activation [18,19]. Meanwhile, constructing a g-C_3_N_4_/ZnCdS heterointerface is also considered an effective strategy for promoting the separation and migration of photogenerated charge carriers [20,21,22,23,24].

It should be noted that most related studies have mainly focused on the individual contribution of defect engineering or heterostructure construction, whereas systematic investigation into their dual synergy in photocatalytic H_2_O_2_ production remains limited [19,20,21,22,23,24]. In this work, g-C_3_N_4_ was coupled with Zn-vacancy-containing ZnCdS to construct a CN/Zn_V_-ZCS composite photocatalyst for visible-light-driven H_2_O_2_ production. The optimized CN/Zn_V_-10 achieved an H_2_O_2_ yield of 44.58 mmol g^−1^ within 60 min under 425 nm LED irradiation and exhibited good cycling stability. Based on the structural characterizations, XPS analysis, photoelectrochemical measurements, and reactive-species results, the enhanced performance is mainly attributed to the synergistic effect of Zn vacancies and the heterointerface: Zn vacancies are favorable for O_2_ adsorption/activation, whereas the CN/Zn_V_-ZCS heterointerface promotes the separation and migration of photogenerated charge carriers. On this basis, a Z-scheme heterojunction is constructed between CN and Zn_V_-ZCS [20,21,22,23,24].

## 2. Materials and Methods

### 2.1. Materials

The chemicals used in this work included zinc acetate dihydrate (Zn(CH_3_COO)_2_·2H_2_O, ≥99.0%, Aladdin, Shanghai, China), cadmium acetate dihydrate (Cd(CH_3_COO)_2_·2H_2_O, ≥99.9%, Aladdin, Shanghai, China), ethylenediamine (C_2_H_8_N_2_, ≥99.0%, Aladdin, Shanghai, China), thioacetamide (TAA, C_2_H_5_NS, ≥99.0%, Macklin, Shanghai, China), L-cysteine (C_3_H_7_NO_2_S, ≥99.0%, Aladdin, Shanghai, China), melamine (C_3_H_6_N_6_, ≥99.0%, Sinopharm, Shanghai, China), and lactic acid (LA, analytical grade, Sinopharm, Shanghai, China). All reagents were purchased from commercial suppliers and used without further purification. Deionized water was used throughout all experiments.

### 2.2. Catalyst Preparation

Preparation of ZnCdS (ZCS)

ZnCdS (ZCS) was synthesized as follows. First, 4 mmol of Zn(CH_3_COO)_2_·2H_2_O was dissolved in a mixed solvent containing 25 mL of ethylenediamine and 25 mL of deionized water, followed by the addition of 6 mmol of Cd(CH_3_COO)_2_·2H_2_O. After the solution bec ame clear, 13 mmol of TAA was added, and the mixture was stirred for 30 min to form a light-yellow suspension. The suspension was then transferred into a 50 mL Teflon-lined stainless-steel autoclave (Nantong Feiyu Biological Technology Co., Ltd., Nantong, China) and heated at 220 °C for 24 h. After naturally cooling to room temperature, the precipitate was collected by centrifugation, washed several times with deionized water and absolute ethanol, and finally dried in a vacuum oven at 60 °C for 12 h to obtain ZCS.

Preparation of Zn_V_-ZnCdS (Zn_V_-ZCS)

ZnV-ZCS was prepared through a modified hydrothermal process. Specifically, 4 mmol of Zn(CH_3_COO)_2_·2H_2_O was dissolved in 30 mL of deionized water, followed by the addition of 6 mmol of Cd(CH_3_COO)_2_·2H_2_O. After the solution became clear, 20 mmol of L-cysteine was added, and the mixture was stirred for 30 min to form a white suspension. The obtained suspension was then transferred to a 50 mL autoclave and heated at 180 °C for 18 h. After reaction, the product was collected by centrifugation, washed repeatedly with deionized water and ethanol, and dried in a vacuum oven at 60 °C for 12 h to obtain Zn_V_-ZCS.

Preparation of g-C_3_N_4_ (CN)

CN was prepared by a thermal polymerization method. Briefly, 1 g of melamine was placed in a covered porcelain crucible and calcined in air at 520 °C for 3 h with a heating rate of 8 °C min^−1^. After natural cooling, the resulting light-yellow solid was ground into powder and denoted as CN.

Preparation of C_3_N_4_/Zn_V_-ZnCdS-10 (CN/Zn_V_-10)

For the preparation of CN/Zn_V_-10, 50 mg of Zn_V_-ZCS was dispersed in 50 mL of deionized water, stirred for 30 min, and ultrasonicated for 3 h to obtain suspension A. Meanwhile, 10 mg of CN was dispersed in 20 mL of deionized water and treated under the same stirring and ultrasonication conditions to obtain suspension B. Suspensions A and B were then mixed and stirred overnight. The resulting precipitate was collected by centrifugation, washed several times with deionized water and ethanol, and dried at 60 °C for 10 h to obtain the yellow powder sample denoted as CN/Zn_V_-10. By changing the amount of CN to 5, 15, and 20 mg under otherwise identical conditions, a series of composite samples, namely CN/Zn_V_-5, CN/Zn_V_-15, and CN/Zn_V_-20, was also prepared. For comparison, the vacancy-free CN/ZCS composite was prepared following the same procedure as that used for CN/Zn_V_-10, except that ZCS was used in place of Zn_V_-ZCS under otherwise identical conditions.

## 3. Results and Discussion

### 3.1. Characterizations of the Samples

**Figure 1:**
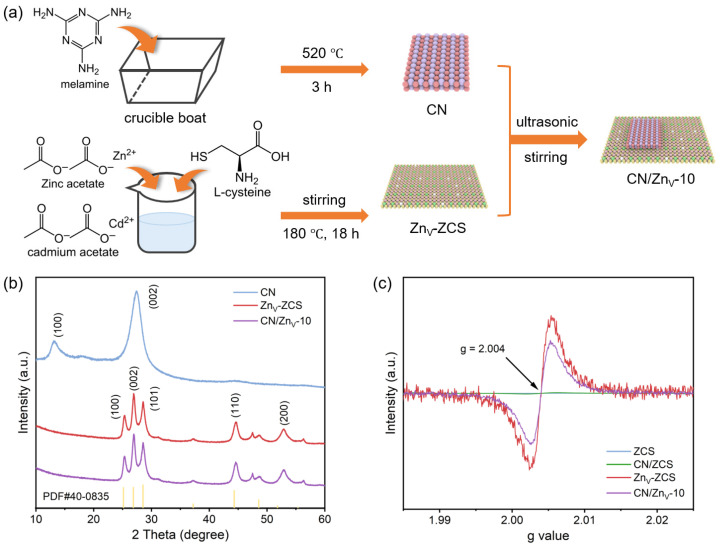
(**a**) Schematic illustration of the fabrication process of the CN/Zn_V_-ZCS composite photocatalyst. (**b**) XRD patterns of CN, Zn_V_-ZCS, and CN/Zn_V_-10. (**c**) EPR spectra of ZCS, CN/ZCS, Zn_V_-ZCS, and CN/Zn_V_-10.

Figure 1a illustrates the fabrication route of the CN/Zn_V_-ZCS composite photocatalyst, in which CN and Zn_V_-ZCS were first synthesized separately and then coupled through ultrasonic dispersion and stirring. Although this strategy is relatively simple, it is favorable for establishing sufficient contact between the two components and thus provides a basis for subsequent interfacial construction. According to Figure 1b, CN exhibits two characteristic diffraction peaks at about 13.1° and 27.4°, corresponding to the in-plane structural ordering and interlayer stacking of g-C_3_N_4_, respectively. The diffraction peaks of Zn_V_-ZCS match well with those of standard ZnCdS, indicating good crystallinity of the prepared sulfide. For CN/Zn_V_-10, the XRD pattern is still dominated by the characteristic peaks of Zn_V_-ZCS, whereas the diffraction signals of CN are relatively weak, which can be attributed to the low CN content and the limited crystallinity of CN itself. Meanwhile, no obvious impurity peaks are observed after compounding, suggesting that no detectable secondary phase is formed. As further evidenced by Figure 1c, pristine ZCS and vacancy-free CN/ZCS show nearly featureless EPR signals, whereas Zn_V_-ZCS exhibits an obvious signal near g = 2.004, which is generally associated with vacancy-related unpaired electrons [19,25]. Importantly, CN/Zn_V_-10 still retains a detectable signal at the same g value, although its intensity is weaker than that of Zn_V_-ZCS, likely due to the dilution effect of CN and the changed local electronic environment after coupling. These results provide direct EPR evidence for the successful introduction of Zn vacancies into Zn_V_-ZCS and further suggest that the vacancy-related electronic features can still be preserved after composite formation. Taken together, these results demonstrate that the target composite has been successfully constructed and that Zn-vacancy engineering indeed introduces defect-related electronic features that are favorable for subsequent O_2_ adsorption/activation and interfacial charge regulation [19,25].

**Figure 2:**
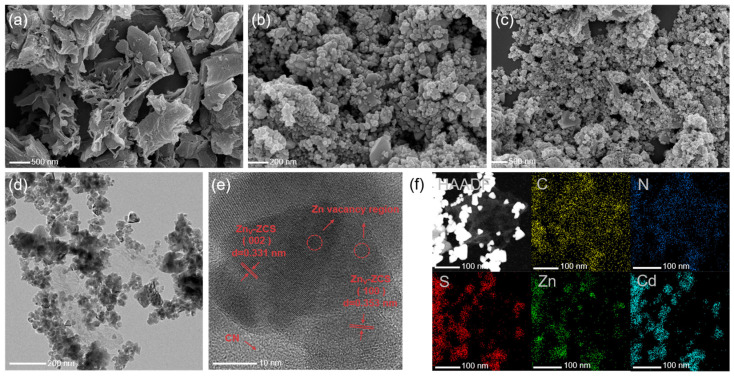
Morphological and structural characterization of the samples: SEM images of (**a**) CN, (**b**) Zn_V_-ZCS, and (**c**) CN/Zn_V_-10; (**d**) TEM image of CN/Zn_V_-10; (**e**) HRTEM image of CN/Zn_V_-10; and (**f**) HAADF-STEM image and corresponding elemental mapping images of C, N, Zn, Cd, and S for CN/Zn_V_-10.

Figure 2 further reveals the morphology and microstructure of the samples. As seen in Figure 2a–c, CN displays a typical wrinkled layered morphology, whereas Zn_V_-ZCS is mainly composed of aggregated nanoparticles. After compounding, CN/Zn_V_-10 still exhibits a particle-dominated morphology that is closer to that of Zn_V_-ZCS, which is consistent with the relatively high proportion of Zn_V_-ZCS and the low loading amount of CN in the composite. The TEM image in Figure 2d shows that the composite is mainly composed of particulate components, while low-contrast regions are tightly attached to the particle surface, indicating that the two phases are not simply physically mixed but instead form close interfacial contact. A closer look at Figure 2e shows clear lattice fringes with spacings of about 0.331 and 0.353 nm, which can be assigned to the (002) and (100) planes of Zn_V_-ZCS, respectively, suggesting that Zn_V_-ZCS maintains good crystallinity after compounding. The HAADF-STEM image and elemental mapping in Figure 2f further confirm the coexistence of C, N, Zn, Cd, and S in the selected region, where C and N are relatively diffuse, while Zn, Cd, and S are mainly concentrated in the particulate regions. Overall, the SEM, TEM, HRTEM, and elemental mapping results consistently indicate that CN and Zn_V_-ZCS are successfully coupled to form an intimate heterointerface, which provides direct structural evidence for the subsequent interfacial separation and migration of photogenerated charge carriers.

**Figure 3:**
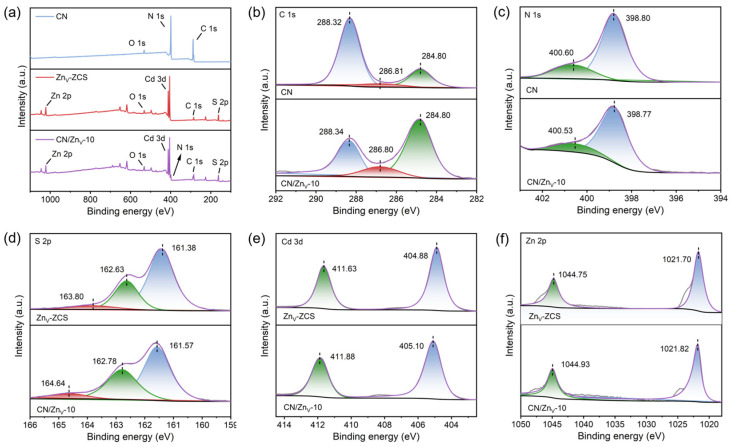
XPS characterization of CN, Zn_V_-ZCS, and CN/Zn_V_-10: (**a**) survey spectra and high-resolution spectra of (**b**) C 1s, (**c**) N 1s, (**d**) S 2p, (**e**) Cd 3d, and (**f**) Zn 2p.

The XPS results of CN, Zn_V_-ZCS, and CN/Zn_V_-10 are summarized in Figure 3. As indicated by Figure 3a, the survey spectrum of CN/Zn_V_-10 simultaneously contains the signals of C, N, Zn, Cd, and S, further confirming the successful coupling of CN and Zn_V_-ZCS. For CN, the main C 1s peak at 288.32 eV in Figure 3b can be assigned to the N–C=N species in the g-C_3_N_4_ framework, while the N 1s peaks at 398.80 and 400.60 eV in Figure 3c correspond to sp^2^-hybridized C–N=C and bridging/amino-related nitrogen species, indicating that the basic CN framework is well preserved after compounding. For Zn_V_-ZCS, the characteristic peaks in Figure 3d–f can be assigned to S^2−^, Cd^2+^, and Zn^2+^ species in metal sulfides. More importantly, compared with the corresponding single components, the N 1s peaks of CN/Zn_V_-10 shift slightly toward lower binding energy, whereas the Zn 2p, Cd 3d, and S 2p peaks shift as a whole toward higher binding energy. For instance, Zn 2p_3/2_ shifts from 1021.70 to 1021.82 eV, Cd 3d_5/2_ from 404.88 to 405.10 eV, and S 2p_3/2_ from 161.38 to 161.57 eV. This cooperative shift, namely the negative shift on the CN side and the positive shift on the Zn_V_-ZCS side, indicates evident interfacial electron redistribution after contact between the two phases and suggests a tendency of electron migration from Zn_V_-ZCS to CN, thereby establishing close electronic coupling at the interface [19,25]. In other words, CN and Zn_V_-ZCS are not simply physically mixed; rather, they form a composite with clear interfacial interaction, which provides an important electronic basis for the subsequent efficient separation and migration of photogenerated charge carriers [19,25].

### 3.2. Photocatalytic H_2_O_2_ Production Performance

**Figure 4:**
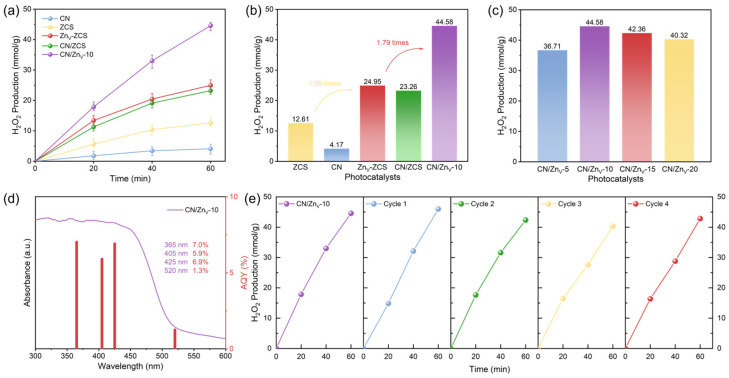
Photocatalytic H_2_O_2_ production performance of the samples under visible-light irradiation: (**a**) time-dependent H_2_O_2_ yields over CN, ZCS, Zn_V_-ZCS, CN/ZCS, and CN/Zn_V_-10; (**b**) comparison of the H_2_O_2_ yields of CN, ZCS, Zn_V_-ZCS, CN/ZCS, and CN/Zn_V_-10 after 60 min; (**c**) effect of CN content on H_2_O_2_ production over CN/Zn_V_-X composites (X = 5, 10, 15, and 20); (**d**) UV–vis absorption profile and AQY values of CN/Zn_V_-10 at different wavelengths; and (**e**) cycling stability of CN/Zn_V_-10. Reaction conditions: 10 mg catalyst, 45 mL deionized water, 5 mL lactic acid, O_2_ bubbling for 15 min before irradiation, and 425 nm LED light source, unless otherwise specified.

Figure 4a shows the photocatalytic hydrogen peroxide (H_2_O_2_) production performances of different samples under visible-light irradiation. As the reaction time increases, the H_2_O_2_ yields of all samples rise continuously, but their catalytic activities differ significantly. CN exhibits the lowest activity, while the H_2_O_2_ yield of ZCS is obviously higher than that of CN. After introducing Zn vacancies, Zn_V_-ZCS shows a further enhanced activity compared with ZCS, indicating that Zn vacancies are favorable for promoting oxygen adsorption and activation. Meanwhile, the vacancy-free CN/ZCS composite also displays remarkably higher activity than pristine ZCS, confirming that heterointerface construction can effectively improve the photocatalytic performance. Among all the tested samples, CN/Zn_V_-10 maintains the highest H_2_O_2_ production capacity throughout the whole reaction stage, reaching an H_2_O_2_ yield of 44.58 mmol g^−1^ after 60 min of reaction, which is approximately 10.69, 3.54, 1.79, and 1.92 times those of CN, ZCS, Zn_V_-ZCS, and CN/ZCS, respectively. As shown in Figure 4b, CN/Zn_V_-10 exhibits the optimal photocatalytic activity among all reference and composite samples. These results demonstrate that both Zn-vacancy engineering and heterointerface construction exert positive effects on photocatalytic H_2_O_2_ production, and the synergistic integration of the two factors in CN/Zn_V_-10 leads to the best performance, revealing an obvious synergistic effect. More specifically, the effect of CN content on the H_2_O_2_ production performance is presented in Figure 4c, where CN/Zn_V_-5, CN/Zn_V_-10, CN/Zn_V_-15, and CN/Zn_V_-20 deliver H_2_O_2_ yields of 36.71, 44.58, 42.36, and 40.32 mmol g^−1^, respectively, indicating the presence of an optimal composite ratio in this system. When the CN content is too low, the interfacial synergy may not be fully developed; when the CN content becomes excessive, the effective light harvesting of Zn_V_-ZCS or the interfacial exposure and charge-transfer efficiency may be partly weakened, resulting in a slight decline in activity. The UV–vis absorption profile and AQY values of CN/Zn_V_-10 at different wavelengths are presented in Figure 4d, and the variation trend of AQY is generally consistent with the light-absorption range of the material, confirming a clear photoresponse of the reaction. In terms of stability, Figure 4e shows that CN/Zn_V_-10 still retains high H_2_O_2_ production ability after five consecutive cycles, demonstrating good stability and reusability. Overall, the superior performance of CN/Zn_V_-10 does not arise from a single factor, but from the synergy between Zn vacancies and the heterointerface: the former is mainly favorable for O_2_ adsorption/activation, whereas the latter mainly promotes the separation and migration of photogenerated charge carriers, and together they enhance photocatalytic H_2_O_2_ production [18,19,23,26,27].

### 3.3. Photoelectrochemical Properties Characterization

**Figure 5:**
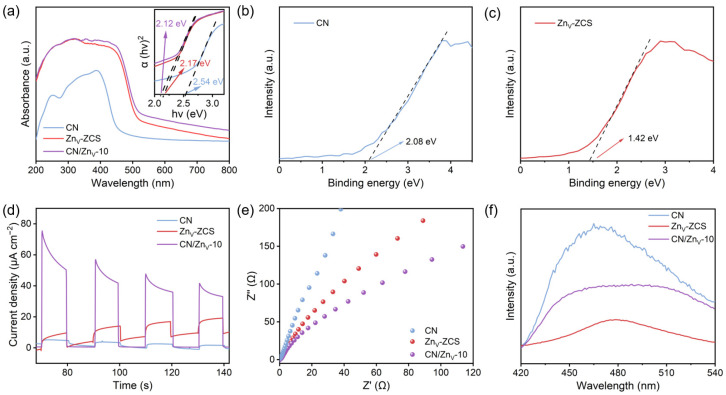
Photoelectrochemical and optical properties of CN, Zn_V_-ZCS, and CN/Zn_V_-10: (**a**) UV–vis diffuse reflectance spectra and the corresponding Tauc plots; valence-band XPS spectra of (**b**) CN and (**c**) Zn_V_-ZCS; (**d**) transient photocurrent responses; (**e**) electrochemical impedance spectroscopy (EIS) Nyquist plots; and (**f**) steady-state photoluminescence (PL) spectra.

To further understand the different photocatalytic H_2_O_2_ production performances of the samples, Figure 5 systematically examines their light-harvesting properties, band structures, and charge-carrier behaviors. As shown in Figure 5a, Zn_V_-ZCS exhibits much stronger visible-light absorption than CN, while the absorption edge of CN/Zn_V_-10 is further extended after compounding, indicating improved visible-light utilization in the composite. The corresponding Tauc plots in Figure 5a suggest that the band gaps of CN, Zn_V_-ZCS, and CN/Zn_V_-10 are about 2.54, 2.17, and 2.12 eV, respectively, indicating that the composite maintains a relatively narrow band gap together with enhanced visible-light response. To more reliably determine the band-edge positions of CN and Zn_V_-ZCS, VB-XPS measurements were further performed, as shown in Figure 5b,c. The valence-band positions versus NHE were calculated according to the following equation:

(1) $$E_{VB,NHE}=\varphi +E_{VB,XPS}-4.44$$

where φ represents the work function of the XPS instrument, which is 4.2 eV in this work. According to the VB-XPS onset values and the above conversion formula, the VB positions of CN and Zn_V_-ZCS are determined to be about 1.84 and 1.18 V versus NHE, respectively. By combining these VB values with the optical band gaps obtained from the Tauc plots, the corresponding conduction-band positions can be determined according to the following equation:

(2) $$E_{CB}=E_{VB}-E_{g}$$

Accordingly, the CB positions of CN and Zn_V_-ZCS are estimated to be about −0.70 and −0.99 V versus NHE, respectively. These results indicate that CN and Zn_V_-ZCS possess distinct band structures, in which Zn_V_-ZCS has a more negative CB position that is favorable for reduction reactions, whereas CN retains a more positive VB position that is beneficial for preserving stronger oxidation ability in the composite system [20,21,22,23,24,28,29,30]. In addition, the Mott–Schottky plots of CN and Zn_V_-ZCS shown in Figure S1 indicate that both materials are n-type semiconductors, with flat-band potentials of about −0.62 and −0.88 V (vs. Ag/AgCl), respectively. These results further support the different electron-energy levels of the two components and provide additional thermodynamic evidence for interfacial electron redistribution and possible directional charge transfer [20,21,22,23,24,28,29,30]. In terms of carrier dynamics, Figure 5d shows that CN/Zn_V_-10 consistently exhibits the highest transient photocurrent response during repeated light on–off cycles, indicating more efficient generation and separation of photogenerated charge carriers. The corresponding EIS Nyquist plots in Figure 5e reveal that CN/Zn_V_-10 has the smallest arc radius, implying the lowest interfacial charge-transfer resistance. Meanwhile, the steady-state PL spectra in Figure 5f show that CN gives the strongest emission, whereas CN/Zn_V_-10 displays markedly quenched PL intensity, suggesting that electron–hole recombination is effectively suppressed after compounding.

Taken together, these results demonstrate that coupling CN with Zn_V_-ZCS not only improves visible-light response but also significantly promotes the separation and migration of photogenerated charge carriers, which is consistent with heterointerface-induced optimization of charge behavior and provides key support for the subsequent discussion of interfacial charge-transfer mechanism [20,21,22,23,24,28,29,30].

### 3.4. Photocatalytic Mechanism Studies

**Figure 6:**
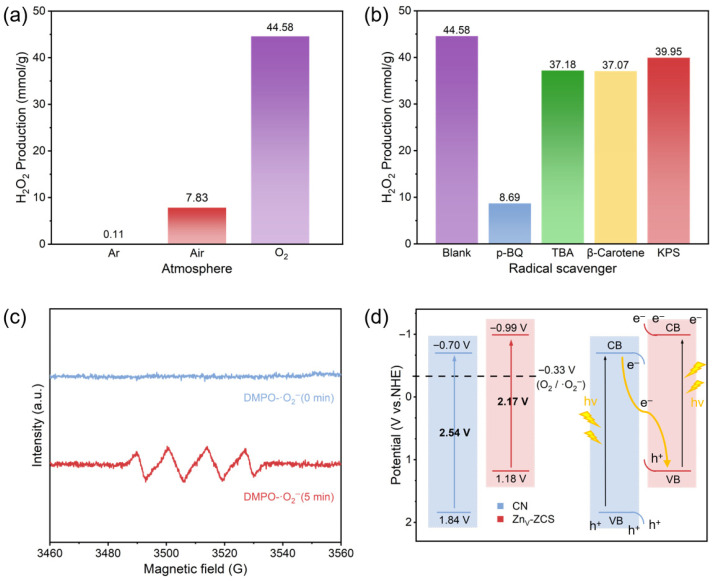
Mechanism studies of photocatalytic H_2_O_2_ production over CN/Zn_V_-10: (**a**) effect of reaction atmosphere (O_2_, air, and Ar) on H_2_O_2_ generation; (**b**) effects of different scavengers on H_2_O_2_ production; (**c**) DMPO−·O_2_^−^ EPR spectra recorded under dark and light-irradiation conditions; and (**d**) schematic band structures of CN and Zn_V_-ZCS before contact, constructed from VB-XPS and optical band-gap results, and the proposed Z-scheme interfacial charge-transfer pathway under visible-light irradiation. Unless otherwise specified, the photocatalytic tests were carried out under the same conditions as those used for H_2_O_2_ production.

To further clarify the reaction pathway of photocatalytic H_2_O_2_ production over CN/Zn_V_-10 and the origin of its enhanced performance, Figure 6 provides mechanistic information from the aspects of reaction atmosphere, reactive-species trapping, radical detection, and band-structure-based interfacial charge-transfer analysis. As shown in Figure 6a, CN/Zn_V_-10 delivers the highest H_2_O_2_ yield under an O_2_ atmosphere, whereas the yield decreases markedly in air and becomes almost negligible under Ar. This result indicates that O_2_ is not only the reactant source for H_2_O_2_ formation, but also the key electron acceptor in this system [1,2,3,4,5,6]. Figure 6b further shows that the addition of p-benzoquinone causes a pronounced decrease in H_2_O_2_ production, while tert-butanol, β-carotene, and potassium persulfate induce only relatively limited inhibition, suggesting that the ·O_2_^−^-related pathway plays a dominant role in the reaction process [11,12,13,14,15,16,31,32]. Consistently, the EPR spectra in Figure 6c display an obvious DMPO-·O_2_^−^ signal under light irradiation, whereas the corresponding signal is very weak in the dark, confirming the generation and participation of superoxide radicals during the reaction [11,12,13,14,15,16,31]. These results collectively indicate that the photocatalytic H_2_O_2_ production over CN/Zn_V_-10 proceeds mainly through a stepwise O_2_ reduction route.

The superior activity of CN/Zn_V_-10 originates from the synergistic effect of Zn-vacancy engineering and heterointerface construction. On the one hand, the intimate coupling between CN and Zn_V_-ZCS, as evidenced by SEM/TEM/HRTEM observations and elemental mapping, together with the systematic XPS binding-energy shifts, demonstrates strong interfacial electronic interaction and electron redistribution after contact. On the other hand, the distinct band-edge structures derived from VB-XPS and optical band-gap analysis, together with the n-type Mott–Schottky behavior of CN and Zn_V_-ZCS, indicate that an internal electric field can be formed at the interface, which is beneficial for directional carrier migration and suppression of charge recombination. This conclusion is further supported by the enhanced transient photocurrent response, the smaller EIS semicircle, and the quenched PL intensity of CN/Zn_V_-10, all of which confirm more efficient charge separation and transfer in the composite [6,20,21,22,23,24,27,28,32,33].

**Figure 7:**
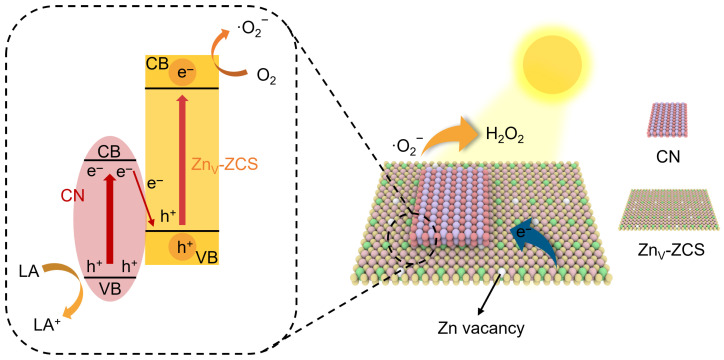
Proposed Z-scheme photocatalytic mechanism for H_2_O_2_ production over the CN/Zn_V_-10 heterostructure.

Further insight is provided by the schematic band structures in Figure 6d. In this work, the band positions of CN and Zn_V_-ZCS were determined by combining the VB-XPS results with the optical band gaps obtained from the Tauc plots. Accordingly, CN and Zn_V_-ZCS possess distinct band-edge structures, in which Zn_V_-ZCS shows a more negative conduction-band position, while CN retains a more positive valence-band position. Before contact, these differences provide the thermodynamic basis for interfacial charge redistribution after coupling. Under visible-light irradiation, both components can be excited, and the interfacial electronic structure is favorable for the recombination of low-energy charge carriers while retaining the electrons with stronger reduction ability and the holes with stronger oxidation ability. This interpretation is consistent with the above XPS, VB-XPS, and photoelectrochemical results, and provides a reasonable explanation for the enhanced charge-separation behavior in the composite system [19,21,23,25]. Based on the available evidence, a Z-scheme interfacial charge-transfer pathway operates in this system [19,21,23,25]. More importantly, Zn vacancies are mainly favorable for O_2_ adsorption/activation, whereas the CN/Zn_V_-ZCS heterointerface mainly promotes the separation and migration of photogenerated charge carriers. Based on these results, the overall photocatalytic mechanism over CN/Zn_V_-10 is summarized in Figure 7. Upon visible-light irradiation, both CN and Zn_V_-ZCS can be photoexcited to generate electron–hole pairs; Zn vacancies mainly facilitate O_2_ adsorption and activation, whereas the heterointerface promotes efficient charge separation and migration, thereby enabling enhanced photocatalytic H_2_O_2_ production.

## 4. Conclusions

In summary, a CN/Zn_V_-ZCS composite photocatalyst was successfully constructed by coupling g-C_3_N_4_ with Zn-vacancy-containing ZnCdS for visible-light-driven H_2_O_2_ production. Structural characterization, morphology analysis, and XPS results collectively demonstrate the successful introduction of Zn vacancies into ZnCdS, the formation of an intimate heterointerface between CN and Zn_V_-ZCS, and evident interfacial electron redistribution after compounding. Under 425 nm LED irradiation, the optimized CN/Zn_V_-10 achieves an H_2_O_2_ yield of 44.58 mmol g^−1^ within 60 min, outperforming pristine CN, ZCS, Zn_V_-ZCS, and vacancy-free CN/ZCS, together with good cycling stability. The newly introduced control samples further confirm that both Zn-vacancy engineering and heterointerface construction contribute positively to H_2_O_2_ production, while their integration in CN/Zn_V_-10 leads to the highest activity. Combined VB-XPS, light-harvesting, transient photocurrent, electrochemical impedance, PL, trapping, and EPR results indicate that Zn vacancies are mainly favorable for O_2_ adsorption/activation, whereas the CN/Zn_V_-ZCS heterointerface mainly promotes the separation and migration of photogenerated charge carriers. Based on the available evidence, a Z-scheme interfacial charge-transfer pathway is established, while the more essential understanding is that defect regulation and interfacial engineering are synergistically integrated in this system. This work provides a feasible strategy for efficient visible-light-driven H_2_O_2_ production through the synergy of defect and interface engineering.

## Data Availability

The original contributions presented in this study are included in the article/Supplementary Material. Further inquiries can be directed to the corresponding authors.

## Supplementary Materials

The following supporting information can be downloaded at: https://www.mdpi.com/article/10.3390/nano16080484/s1, Figure S1: Mott–Schottky plots of (a) CN and (b) ZnV-ZCS measured at different frequencies; Table S1: XPS-derived surface atomic contents of CN; Table S2: XPS-derived surface atomic contents of ZnV-ZCS; Table S3: XPS-derived surface atomic contents of CN/Zn_V_-10.

## References

[1] Jiang Z., Li C., Qi F., Wang Z., Liu Y., Li F., Wang H., Bian Z., Zhu M., Kumirska J. (2025). A Review on Photocatalytic Hydrogen Peroxide Production from Oxygen: Material Design, Mechanisms, and Applications. ACS Appl. Mater. Interfaces.

[2] Guo Y., Tong X., Yang N. (2023). Photocatalytic and Electrocatalytic Generation of Hydrogen Peroxide: Principles, Catalyst Design and Performance. Nano-Micro Lett..

[3] Khan S., Qaiser M.A., Qureshi W.A., Haider S.N.-u.-Z., Yu X., Wang W., Liu Q. (2024). Photocatalytic hydrogen peroxide production: Advances, mechanistic insights, and emerging challenges. J. Environ. Chem. Eng..

[4] Yang Y., Wang C., Li Y., Liu K., Ju H., Wang J., Tao R. (2024). Porous organic framework materials for photocatalytic H_2_O_2_ production. J. Mater. Sci. Technol..

[5] Wang W., Wang X., Gao M., Li Z., Zhou W. (2024). Microenvironmental regulation of covalent organic frameworks for photocatalytic hydrogen peroxide production. Coord. Chem. Rev..

[6] Li X., Wan Y., Deng F., Zhou Y., Chen P., Dong F., Jiang J. (2025). Advances in Z-scheme and S-scheme heterojunctions for photocatalytic and photoelectrocatalytic H_2_O_2_ production. Chin. Chem. Lett..

[7] Qin J., Li J., Chu K., Yang G., Zhang L., Xia X., Xuan P., Chen X., Weng B., Huang H. (2024). Biomimetic Solar Photocatalytic Reactor for Selective Oxidation of Aromatic Alcohols with Enhanced Solar-Energy Utilization. Adv. Funct. Mater..

[8] Pei J., Li H., Yu D., Zhang D. (2024). g-C_3_N_4_-Based Heterojunction for Enhanced Photocatalytic Performance: A Review of Fabrications, Applications, and Perspectives. Catalysts.

[9] Ahmed M.A., Mahmoud S.A., Mohamed A.A. (2024). Unveiling the photocatalytic potential of graphitic carbon nitride (g-C_3_N_4_): A state-of-the-art review. RSC Adv..

[10] Liu P., Liang T., Li Y., Zhang Z., Li Z., Bian J., Jing L. (2024). Photocatalytic H_2_O_2_ production over boron-doped g-C_3_N_4_ containing coordinatively unsaturated FeOOH sites and CoO_x_ clusters. Nat. Commun..

[11] Nie L., Chen H., Wang J., Yang Y., Fang C. (2024). Enhanced Visible-Light H_2_O_2_ Production over Pt/g-C_3_N_4_ Schottky Junction Photocatalyst. Inorg. Chem..

[12] Zhang Q., Wang B., Miao H., Fan J., Sun T., Liu E. (2024). Efficient photocatalytic H_2_O_2_ production over K^+^-intercalated crystalline g-C_3_N_4_ with regulated oxygen reduction pathway. Chem. Eng. J..

[13] Mandal R., Bhattacharyya S. (2024). Integrating a Dual Mode of Intrinsic Structural Modifications of g-C_3_N_4_ by K^+^ Ions and Amorphous Carbon for Efficient Photocatalytic H_2_O_2_ Production. J. Phys. Chem. C.

[14] Jiang J., Chen Y., Zhou S., Xie H., Li C., Wei Z., Kong Y. (2024). Dual defect sites at g-C_3_N_4_ synergistically induce the electron localization effect for boosting photocatalytic H_2_O_2_ production. Catal. Sci. Technol..

[15] Sun Y., Wang D., Yang Y., Zhao Q., Yang S., Luo X., Zhao Q., Zhang J.Z. (2024). Enhanced solar-light driven H_2_O_2_ production with g-C_3_N_4_ nanosheets by defect engineering. Surf. Interfaces.

[16] Wang T., Xin J., Li Z., Fan Y., Wang Y. (2023). Application of single-atom Ti-doped g-C_3_N_4_ in photocatalytic H_2_O_2_ production. Mater. Adv..

[17] Wang X., Liu B., Ma S., Zhang Y., Wang L., Zhu G., Huang W., Wang S. (2024). Induced dipole moments in amorphous ZnCdS catalysts facilitate photocatalytic H_2_ evolution. Nat. Commun..

[18] Peng H., Yang H., Han J., Liu X., Su D., Yang T., Liu S., Pao C.-W., Hu Z., Zhang Q. (2023). Defective ZnIn_2_S_4_ Nanosheets for Visible-Light and Sacrificial-Agent-Free H_2_O_2_ Photosynthesis via O_2_/H_2_O Redox. J. Am. Chem. Soc..

[19] Wang H., Wang X., Hu P., Liu T., Weng B., Ye K.-H., Luo Y., Ji H. (2024). Vacancy pair induced surface chemistry reconstruction of Cs_2_AgBiBr_6_/Bi_2_WO_6_ heterojunction to enhance photocatalytic CO_2_ reduction. Appl. Catal. B Environ. Energy.

[20] Yang Y., Li Y., Ma X., Xie L., Lv D., Jiang L., He J., Chen D., Wang J. (2023). Direct Z-scheme WO_3_/covalent organic framework (COF) heterostructure for enhanced photocatalytic hydrogen peroxide production in water. Catal. Sci. Technol..

[21] Gao J., Yang S., Wei P., Wang S., Zheng L., Lan L., Wang Y., Li Y., Chen C., He G. (2025). Enhanced photocatalytic H_2_O_2_ production through synergistic double-vacancy engineering in Z-scheme TiO_2−x_/g-C_3_N_4−x_ heterojunction. Sep. Purif. Technol..

[22] Zhang T., Zhang T., Fu H., Feng L., Zhang Q., Ren S., Cheng J., Liang Q., Xiao X. (2024). Effective enhanced photocatalytic H_2_O_2_ production through synergistic oxygen vacancy defects and In situ-growth Z-scheme BiOBr/WO_3_ heterojunction with compact interface. J. Solid State Chem..

[23] Che Y., Wang K., Wang C., Weng B., Chen S., Meng S. (2026). Lattice match-enabled Zn_3_In_2_S_6_@CdS S-scheme heterojunction with S covalent bond bridge for simultaneous H_2_O_2_ photosynthesis and H_2_ production. J. Mater. Sci. Technol..

[24] Feng X., Liu J., Zheng J., Luo Y., Cai W., Liao Z., Fang Y. (2024). Z-Scheme g-C_3_N_4_-Au-MoO_3−x_ heterojunction for boosted photocatalytic H_2_O_2_ production and enhanced selective oxidation of cyclohexane. J. Organomet. Chem..

[25] Xia S., Yuan Z., Meng Y., Zhang C., Li X., Ni Z., Zhang X. (2024). Fabrication of site activated and synergistic double vacancy ZnIn_2_S_4_ for highly efficient bifunctional photocatalysis: Nitrogen reduction and oxidative degradation. J. Mater. Chem. A.

[26] Meng L., Zhao C., Zhang X., Guo R., Zheng Y., Chu H., Fu H., Wang P., Wang C.-C. (2024). Piezo-photocatalytic synergetic for H_2_O_2_ generation via dual-pathway over Z-scheme ZIF-L/g-C_3_N_4_ heterojunction. Nano Energy.

[27] Ma R., Li S., Xiong Y., Wang J., Guan X., Li J., Zheng X. (2025). Synergistic promotion photocatalytic H_2_O_2_ production over modified g-C_3_N_4_ via defect and doping engineering. Appl. Catal. A Gen..

[28] Hou L., Li W., Wu Z., Wei Q., Yang H., Jiang Y., Wang T., Wang Y., He Q. (2022). Embedding ZnCdS@ZnIn_2_S_4_ into thiazole-modified g-C_3_N_4_ by electrostatic self-assembly to build dual Z-scheme heterojunction with spatially separated active centers for photocatalytic H_2_ evolution and ofloxacin degradation. Sep. Purif. Technol..

[29] Du X., Hu J., Sun Q., Fu H., Zhang J., Chang J., Gao H., Liao Y. (2024). Rapid microwave preparation of CuS/ZnCdS Z-scheme heterojunction for efficient photocatalytic hydrogen evolution. Int. J. Hydrogen Energy.

[30] Abd Rahim I.H., Lee X.Y., Alotabi A.S., Osborn D.J., Adhikari S.G., Andersson G.G., Metha G.F., Adnan R.H. (2024). Photocatalytic H_2_O_2_ production over photocatalysts prepared by phosphine-protected Au101 nanoparticles on WO_3_. Catal. Sci. Technol..

[31] Tran D.D., Vuong H.-T., Nguyen D.-V., Ly P.P., Phan P.D.M., Khoi V.H., Mai P.T., Hieu N.H. (2023). Revisiting the roles of dopants in g-C_3_N_4_ nanostructures for piezo-photocatalytic production of H_2_O_2_: A case study of selenium and sulfur. Nanoscale Adv..

[32] Zhou H., Liu R., Xu Y. (2024). A comparative study of H_2_O_2_ production over g-C_3_N_4_ photocatalysts made from different sources. J. Mol. Struct..

[33] Zhang W., Huang Z., Zhang L., Meng Y., Ni Z., Tang H., Xia S. (2023). Construction of zinc-oxygen double vacancies BiOCl/ZnS Z-scheme heterojunction and photocatalytic degradation of norfloxacin. J. Environ. Chem. Eng..

