# Supplementary material for: Synergistic Enhancement of Visible-Light-Driven Photocatalytic H2O2 Production over g-C3N4/ZnCdS by Zn Vacancies and Heterointerface Engineering
Source: Nanomaterials (Basel). 2026 Apr 18;16(8):484. doi: 10.3390/nano16080484 (PMC13118740; doi:10.3390/nano16080484)
Supplement: Supplementary file 1 [file nanomaterials-16-00484-s001.zip › nanomaterials-4254030-supplementary.pdf]

Synergistic Enhancement of Visible-Light-Driven Photocatalytic H_2O_2 Production over g-C_3N_4/ZnCdS by Zn Vacancies and Heterointerface Engineering

Supporting Information

Zhenyu Wang,[a,b] Wei Yan,[a,b] Yingcong Wei,[a,b] Jing Xu,*[a,b] Yuee Xie,*[a,b] Yuanping Chen [a,b] and Xiaohong Yan [b,c]

a. School of Physics and Electronic Engineering, Jiangsu University, Zhenjiang, 212013, Jiangsu, China. E-mail: xjing@ujs.edu.cn, yueex@ujs.edu.cn

b. Quantum Sensing and Agricultural Intelligence Detection Engineering Center of Jiangsu Province, Zhenjiang, 212013, Jiangsu, China

c. School of Science, Nanjing University of Posts and Telecommunications, Nanjing, 210023, Jiangsu, China

1. Catalyst characterization

X-ray diffraction (XRD) patterns were recorded on a **Bruker D2 Phaser diffractometer (Bruker, Karlsruhe, Germany)** using Cu Kα radiation (λ = 1.5418 Å) at 40 kV and 40 mA. Data were collected in the 2θ range of 10–80° with a step size of 0.02° and a scan speed of 2° min^{-1}.

Scanning electron microscopy (SEM) images were obtained on a **JEOL JSM-7001F field-emission scanning electron microscope (JEOL, Tokyo, Japan)** at an accelerating voltage of 15 kV. Secondary-electron (SE) images were mainly used, and backscattered-electron (BSE) images were collected when compositional contrast was required.

Transmission electron microscopy (TEM) images and energy-dispersive X-ray spectroscopy (EDS) elemental mapping were acquired on a **JEOL JEM-2100 transmission electron microscope (JEOL, Tokyo, Japan)** operated at 200 kV. EDS mapping was performed with a probe current of approximately 0.8 nA and a dwell time of approximately 5 μs per pixel.

X-ray photoelectron spectroscopy (XPS) measurements were carried out on a **Thermo Scientific K-Alpha X-ray photoelectron spectrometer (Thermo Fisher Scientific, East Grinstead, UK)** with Al Kα radiation (hν = 1486.6 eV). Survey spectra were recorded with a pass energy of 150 eV, and high-resolution spectra were collected with a pass energy of 20 eV. All binding energies were calibrated using the C 1s peak at 284.80 eV as the reference. XPS data were processed using Avantage software with a Shirley background and Gaussian-Lorentzian peak shapes for fitting. The surface atomic contents of the detected elements were estimated from the XPS survey spectra using the built-in sensitivity-factor-based quantification method in Avantage.

Valence-band XPS (VB-XPS) spectra were recorded on the same **Thermo Scientific K-Alpha X-ray photoelectron spectrometer (Thermo Fisher Scientific, East Grinstead, UK)** using Al Kα radiation. The valence-band onset positions were determined by linear extrapolation of the leading edge of the VB-XPS spectra. The valence-band positions versus the normal hydrogen electrode (NHE) were calculated according to Equation (S1):

$$E_{VB,NHE} = \varphi + E_{VB,XPS} - 4.44 \quad \text{(S1)}$$

where φ is the work function of the XPS instrument, taken as 4.2 eV in this work. The conduction-band positions were then estimated from the optical band gaps according to Equation (S2):

$$E_{CB} = E_{VB} - E_g \quad \text{(S2)}$$

where E_g is the band-gap energy obtained from the Tauc plots. On this basis, the band positions used in Figure 5 and Figure 6d were constructed by combining the VB-XPS results with the optical band-gap values.

Diffuse reflectance UV–vis spectra (DRS) were measured on a **Shimadzu UV-2550 spectrophotometer (Shimadzu, Kyoto, Japan)** equipped with an integrating sphere, using $BaSO_4$ as the reference. The reflectance data were converted into the Kubelka–Munk function for band-gap estimation.

Photoluminescence (PL) spectra were collected on a **JASCO FP-6500 fluorescence spectrophotometer (JASCO, Tokyo, Japan)** with an excitation wavelength of 325 nm and slit widths of 5 nm for both excitation and emission.

Photoelectrochemical measurements were performed on a **CHI660B electrochemical workstation (CH Instruments, Austin, TX, USA)** in a conventional three-electrode system with a Pt plate as the counter electrode and an Ag/AgCl (saturated KCl) electrode as the reference electrode. The working electrodes were prepared by drop-casting a catalyst suspension (5 mg catalyst dispersed in 1 mL ethanol) onto fluorine-doped tin oxide (FTO) glass with an active area of 1.76 cm^2, followed by drying at 60 °C. The resulting catalyst loading was approximately 2.84 mg cm^{-2}. Transient photocurrent responses were recorded under chopped AM 1.5G-simulated illumination (300 W Xe lamp with an AM 1.5G filter, 100 mW cm^{-2}) using 10 s light-on/10 s light-off cycles at 0 V vs. Ag/AgCl. Electrochemical impedance spectroscopy (EIS) measurements were conducted at open-circuit potential with an AC amplitude of 10 mV over the frequency range from 100 kHz to 0.1 Hz in 0.1 M Na_2SO_4 electrolyte.

To evaluate the wavelength-dependent photocatalytic efficiency of CN/ZnV-10, the apparent quantum yield (AQY) was calculated according to Equation (S3):

$$AQY(\%) = \frac{N_{reacted\ electrons}}{N_{incident\ photons}} \times 100 = \frac{n_{product} \times e^{-} per\ product}{N_{photons}} \times 100 \quad \text{(S3)}$$

2. Photocatalytic H_2O_2 production

Photocatalytic H_2O_2 production was carried out in a 100 mL three-necked round-bottom flask at room temperature under magnetic stirring. In a typical experiment, 10 mg of catalyst was dispersed in a mixed solution containing 45 mL of deionized water and 5 mL of lactic acid, where lactic acid served as the sacrificial agent. Before irradiation, the suspension was ultrasonicated and stirred to ensure uniform dispersion of the catalyst. Unless otherwise specified, all photocatalytic tests were performed under identical conditions.

Prior to light irradiation, O_2 was bubbled through the suspension for 15 min to establish an oxygen-rich reaction atmosphere. The photocatalytic reaction was then conducted under irradiation from a 425 nm LED light source for 60 min. Liquid samples were withdrawn at 20 min intervals, and each collected suspension was immediately filtered through a disposable membrane filter to remove catalyst particles before analysis.

The amount of H_2O_2 produced was determined by the iodometric method. Specifically, 50 μL of the filtrate was mixed with 0.5 mL of 0.1 M potassium hydrogen phthalate solution and 0.5 mL of 0.4 M KI solution, followed by standing for 30 min under acidic conditions for full color development. In this process, H_2O_2 oxidizes I^- to I_3^-, which exhibits a characteristic absorption band at around 350 nm. The absorbance was measured by UV–vis spectrophotometry, and the H_2O_2 concentration was calculated according to the corresponding calibration curve.

For the atmosphere-dependent experiments, the reaction system was purged with the corresponding gas (O_2, air, or Ar) before irradiation. For the reactive-species trapping experiments, the designated scavenger was added to the reaction suspension before light irradiation, while all other experimental conditions were kept unchanged. Unless otherwise stated, the H_2O_2 yields reported in this work were obtained after 60 min of visible-light irradiation.

3. Figures and tables

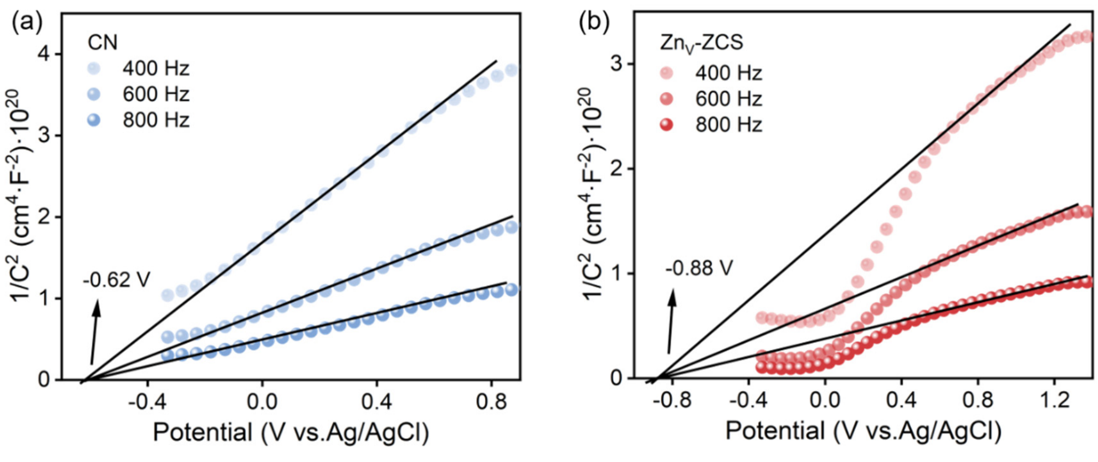

Figure S1. Mott–Schottky plots of (a) CN and (b) Zn$_V$-ZCS measured at different frequencies

Table S1. XPS-derived surface atomic contents of CN.

Element	Atomic percentage (at.%)
C	46.35
N	53.65

Table S2. XPS-derived surface atomic contents of Zn_V-ZCS.

Element	Atomic percentage (at.%)
C	34.51
Cd	20.73
Zn	10.68
S	34.18

Table S3. XPS-derived surface atomic contents of CN/Zn_V-10.

Element	Atomic percentage (at.%)
C	51.26
N	14.29
Cd	10.56
Zn	6.11
S	17.78
